# Coronary computed tomography angiography improves assessment of patients with acute chest pain and inconclusively elevated high-sensitivity troponins

**DOI:** 10.1007/s00330-024-10930-1

**Published:** 2024-08-16

**Authors:** Murat Arslan, Jeroen Schaap, Bart van Gorsel, Anton Aubanell, Ricardo P. J. Budde, Alexander Hirsch, Martijn W. Smulders, Casper Mihl, Peter Damman, Olga Sliwicka, Jesse Habets, Eric A. Dubois, Admir Dedic

**Affiliations:** 1 Department of Cardiology, Erasmus Medical Centre, University Medical Centre Rotterdam, Rotterdam, The Netherlands; 2 Department of Radiology and Nuclear Medicine, Erasmus Medical Centre, University Medical Centre Rotterdam, Rotterdam, The Netherlands; 3 Department of Cardiology, Amphia Hospital, Breda, The Netherlands; 4 Department of Cardiology, Maastricht University Medical Centre, Maastricht, The Netherlands; 5 Department of Radiology, Maastricht University Medical Centre, Maastricht, The Netherlands; 6 Department of Cardiology, Radboud University Medical Centre, Nijmegen, The Netherlands; 7 Department of Radiology, Nuclear Medicine and Anatomy, Radboud University Medical Centre, Nijmegen, The Netherlands; 8 Department of Radiology, Haaglanden Medical Centre, The Hague, The Netherlands; 9 Department of Intensive Care, Erasmus Medical Centre, University Medical Centre Rotterdam, Rotterdam, The Netherlands; 10 Department of Cardiology, Noordwest Group, Alkmaar, The Netherlands

**Keywords:** Computed tomography angiography, Troponin, Non-ST elevated myocardial infarction, Acute coronary syndrome

## Abstract

**Objectives:**

To determine whether coronary computed tomography angiography (CCTA) can improve the diagnostic work-up of patients with acute chest pain and inconclusively high-sensitivity troponins (hs-troponin).

**Methods:**

We conducted a prospective, blinded, observational, multicentre study. Patients aged 30–80 years presenting to the emergency department with acute chest pain and inconclusively elevated hs-troponins were included and underwent CCTA. The primary outcome was the diagnostic accuracy of ≥ 50% stenosis on CCTA to identify patients with type-1 non-ST-segment elevation acute coronary syndrome (NSTE-ACS).

**Results:**

A total of 106 patients (mean age 65 ± 10, 29% women) were enrolled of whom 20 patients (19%) had an adjudicated diagnosis of type-1 NSTE-ACS. In 45 patients, CCTA revealed non-obstructive coronary artery disease (CAD) or no CAD. Sensitivity, specificity, negative predictive value (NPV), positive predictive value and area-under-the-curve (AUC) of ≥ 50% stenosis on CCTA to identify patients with type 1 NSTE-ACS, was 95% (95% confidence interval: 74–100), 56% (45–68), 98% (87–100), 35% (29–41) and 0.83 (0.73–0.94), respectively. When only coronary segments with a diameter ≥ 2 mm were considered for the adjudication of type 1 NSTE-ACS, the sensitivity and NPV increased to 100%. In 8 patients, CCTA enabled the detection of clinically relevant non-coronary findings.

**Conclusion:**

The absence of ≥ 50% coronary artery stenosis on CCTA can be used to rule out type 1 NSTE-ACS in acute chest pain patients with inconclusively elevated hs-troponins. Additionally, CCTA can help improve the diagnostic work-up by detecting other relevant conditions that cause acute chest pain and inconclusively elevated hs-troponins.

**Clinical relevance statement:**

Coronary CTA (CCTA) can safely rule out type 1 non-ST-segment elevation acute coronary syndrome (NSTE-ACS) in patients presenting to the ED with acute chest pain and inconclusively elevated hs-troponins, while also detecting other relevant non-coronary conditions.

**Trial registration:**

Clinicaltrials.gov (NCT03129659). Registered on 26 April 2017

**Key Points:**

- *Acute chest discomfort is a common presenting complaint in the emergency department.*
- *CCTA achieved very high negative predictive values for type 1 NSTE-ACS in this population.*
- *CCTA can serve as an adjunct for evaluating equivocal ACS and evaluates for other pathology.*

**Graphical Abstract:**

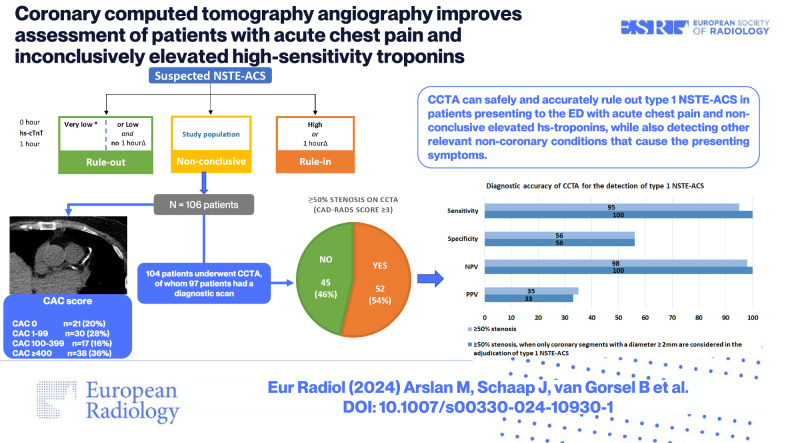

## Introduction

Acute chest discomfort is a very common cause of emergency department (ED) attendance [1]. The underlying reason for these complaints may vary from non-cardiac and often benign causes to life-threatening disorders, such as non-ST-segment elevation acute coronary syndrome (NSTE-ACS) [2]. Currently, diagnostic evaluation at the ED consists of a clinical examination, electrocardiogram (ECG) and measurement of cardiac biomarkers, more precisely high-sensitivity troponins (hs-troponin). Rule-out and rule-in algorithms with hs-troponins are recommended in the management of patients with acute chest pain [2].

Using these algorithms, a considerable number, i.e., 20–30%, of patients do not qualify for rule-out or rule-in and fall into the ‘inconclusively’ category [3, 4]. They represent a heterogeneous group with an unfavourable prognosis [5]. NSTE-ACS still may be their underlying condition and should be excluded. Invasive coronary angiography (ICA), which is considered the reference standard for the detection of obstructive coronary artery disease (CAD), carries inherent risks. In recent years, coronary computed tomography angiography (CCTA) has emerged as a useful non-invasive alternative and can serve as a gatekeeper for ICA, thereby reducing the need for unnecessary invasive procedures [6]. The aim of this study is to investigate whether CCTA can improve the diagnostic work-up of patients with acute chest pain and low-range positive hs-troponins.

## Methods and analysis

### Study design

The study was approved by the local institutional review board (Medical Research Ethics Committee of Erasmus Medical Centre in Rotterdam, the Netherlands, registration number MEC-2017-506) and is registered at clinicaltrials.gov (NCT03129659). All patients provided written informed consent. The study was conducted according to the principles of the Declaration of Helsinki (10th version, October 2013) and in accordance with the Medical Research Involving Human Subjects Act. The Coronary CT Angiography for Improved Assessment of Patients with Acute Chest Pain and Inconclusively Elevated High-Sensitivity troponins (COURSE) study is a prospective, double-blind, observational, multicentre study. The rationale for and design of the trial have been published previously [7]. Patients were enrolled at 3 university hospitals and 1 community hospital in the Netherlands between February 2018 and May 2021. Enrolment was performed during working hours.

### Study population

**Fig. 1:**
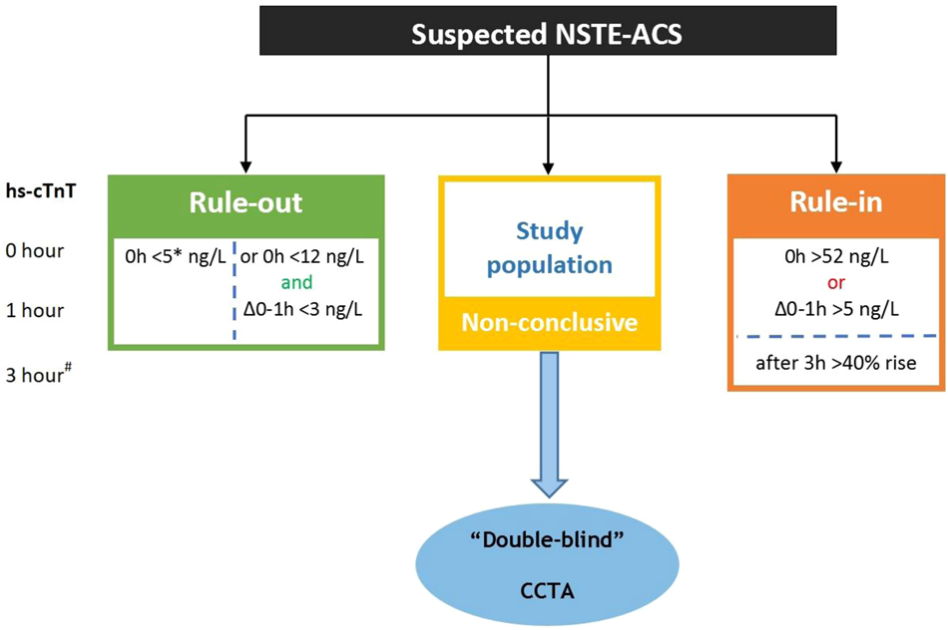
Study population. Inconclusively refers to patients who do not meet the criteria for rule-out or rule-in of the European Society of Cardiology 0-h/1-h algorithm using high-sensitivity cardiac troponins. 0-h/1-h algorithm cut-off levels shown (in ng/L) are specific to the hs-cTnT assay (elecsys; Roche). *Only applicable if chest pain onset > 3 h. ^#^‘0-h/3-h’ algorithm is only used as a substitute in cases where the standard ‘0-h/1-h’ algorithm is not feasible. 0 h, 1 h and 3 h refer to the time (in hours) from the first blood draw. CCTA, coronary computed tomography angiography; hs-cTnT, high-sensitivity cardiac Troponin T; NSTE-ACS, non-ST-segment elevation acute coronary syndrome; ∆, delta

Patients aged 30–80 years presenting to the ED with acute chest pain suspected of NSTE-ACS and inconclusively elevated hs-troponins who did not fulfill criteria for either ‘rule-out’ or ‘rule-in’ of NSTE-ACS were eligible for inclusion (Fig. 1). Exclusion criteria were history of proven CAD, defined as documented prior myocardial infarction, percutaneous coronary intervention or coronary artery bypass graft surgery; previous examination with either ICA or CCTA in the last 3 years; clinical instability, defined as clinical heart failure, hemodynamic instability and severe chest pain; inability or unwillingness to provide informed consent; and a contra-indication for CCTA. Contraindications for CCTA included allergy to iodine contrast media; pregnancy; impaired renal function, defined as estimated glomerular filtering rate < 45 mL/min; severe arrhythmia likely to affect image interpretation; body mass index > 40 kg/m^2^ or inability to cooperate during the examination. Patients included in the study underwent CCTA either at the ED or at the outpatient clinic within 1 week of index presentation.

### Cardiac CT

A non-enhanced scan was performed prior to CCTA in order to determine the coronary artery calcium (CAC) score. Irrespective of CAC scan results, an ECG-triggered, contrast-enhanced CT scan to image the coronary artery lumen and detect the presence of obstructive CAD was performed. The default scan protocol was a prospectively ECG-triggered axial CT scan protocol. Retrospective gating was used in patients with an irregular heart rate. Oral or intravenous metoprolol was given shortly before the scan if deemed necessary. Sublingual nitroglycerin was administered in all patients for vasodilation a few minutes before the scan, in the absence of contraindications. Supplementary Table 1 provides additional information on CT scanners and relevant protocols used in the Course trial.

After the scan was performed the supervising CT reader, at the local hospital, provided a preliminary report. The treating clinician and patient were blinded to the CT results, except in the case of important findings. The results were classified into the following groups:

1. CCTA revealed findings that did not mandate unblinding of the results.
2. CCTA revealed coronary findings with potentially important prognostic implications (significant left main artery disease, significant proximal left anterior descending artery disease, significant three-vessel disease). Significant CAD was defined as ≥ 50% stenosis.
3. CCTA revealed other cardiac (non-coronary) findings that have important prognostic implications
4. CCTA revealed significant non-cardiac findings warranting further management or follow-up.

Findings that were classified into groups two, three and four were revealed to the treating physician and patient. All CT scans were systematically read by one experienced CT reader (dedicated cardiovascular radiologist with > 2 years of clinical experience) at Erasmus Medical Centre at a later stage for final reading. The findings were reported using the Coronary Artery Disease Reporting and Data System (CAD-RADS) [8].

### Clinical endpoints

The primary outcome was the diagnostic accuracy of ≥ 50% stenosis on CCTA to identify patients with type 1 NSTE-ACS, caused by atherothrombotic CAD and usually precipitated by atherosclerotic plaque disruption (rupture or erosion), in terms of sensitivity and negative predictive value (NPV). The secondary outcome was the diagnostic accuracy of ≥ 70% stenosis for all coronary segments, except left main (≥ 50% stenosis), on CCTA to identify patients with type 1 NSTE-ACS, in terms of sensitivity and NPV. Additionally, the occurrence of all-cause mortality and coronary revascularization within 30 days of follow-up was determined.

### Reference standard: adjudicated final diagnosis

The diagnosis of type 1 NSTE-ACS was established by consensus of two independent cardiologists according to current guidelines using all available clinical information including initial clinical presentation, ECG changes, serial laboratory results, (non)-invasive testing and information from the 30-day follow-up [9]. Additional (non)-invasive diagnostic testing and further treatment were performed at the discretion of the treating physician. Because of ethical considerations, patients did not undergo standard ICA to determine the final diagnosis if not deemed necessary by the treating physician. Results of CCTA were not disclosed to the two independent cardiologists who established the definitive clinical diagnosis.

### Sample size calculation

Previous studies show that approximately 40% of all patients with a inconclusively diagnostic work-up eventually turn out to have NSTE-ACS [5]. Based on these observations we assumed that the pre-test probability of NSTE-ACS would be 40% in this population and that a total of 240 patients were required to demonstrate a NPV of 97% with a lower margin of 90%, considering an *α* = 0.05 and *β* = 0.8 and a drop-out rate of 10%.

### Statistical analysis

Results were reported as mean with standard deviation and median with interquartile ranges as appropriate for continuous variables and as numbers and percentages (%) for categorical variables. In addition, the area under the curve (AUC), sensitivity, specificity, NPV and positive predictive value (PPV) were calculated with their corresponding 95% confidence intervals to assess the performance of CCTA. Patients with non-diagnostic CCTAs were excluded from primary diagnostic accuracy analyses of CCTA.

## Results

### Study population

Between February 2018 and May 2021, a total of 106 patients presenting to the ED with acute chest pain, a normal or non-diagnostic ECG and inconclusively elevated hs-troponins were enrolled at 4 centres in the study. The 30-day follow-up was complete in all patients. Table 1 shows patient demographics, clinical characteristics and medical treatment at baseline. The mean age of the study population was 65 ± 10 years, and 31 (29%) patients were women.

**Table 1:** Baseline characteristics

		Study cohort (*N* = 106)
Age, years		65 ± 10
Female sex		31 (29)
BMI, kg/m^2^		27 [24–31]
Risk factors		
	Current smoking	25 (24)
	History of smoking	36 (34)
	Hypertension	58 (55)
	Dyslipidemia	50 (47)
	Diabetes mellitus	27 (26)
	Family history for CAD	37 (35)
	PAD	7 (7)
	Prior stroke/TIA	11 (10)
Medication at presentation		
	Aspirin	14 (13)
	Oral anticoagulation^c^	12 (11)
	ACE inhibitor or ARB	45 (43)
	P2Y12 inhibitor	7 (7)
	Statin	39 (37)
	Beta-blocker	25 (24)
	Diuretics	26 (25)
	CCB	21 (20)
Chest pain symptoms^a^		
	Typical angina Atypical angina Non-anginal chest pain	27 (26) 51 (48) 28 (26)
Blood pressure (mmHg)		
	Systolic	151 ± 26
	Diastolic	82 ± 14
ECG		
	Atrial fibrillation	5 (5)
	Heart rate (per min)	74 ± 14
	Left ventricular hypertrophy	9 (9)
	AV conduction abnormality	7 (7)
	Intraventricular conduction abnormality	19 (18)
	ST-segment deviation^b^	16 (15)
	T wave inversion	19 (18)
Laboratory		
	Time from chest pain onset to first blood draw (hours)	7 [3–17]
	First Hs-cTnT measurement	16 [13–21]
	Second Hs-cTnT measurement	17 [14–22]
	Creatinine clearance, mL/min/m^2^	81 [72–94]
Risk scores		
	HEART score 0–3 (low-risk) 4–6 (intermediate risk) 7–10 (high-risk)	23 (22) 65 (61) 18 (17)
	GRACE score	95 ± 21
	TIMI score	2 [1–3]

Values are mean ± SD, median [interquartile ranges] or *n* (%)

*ACE* angiotensin-converting-enzyme, *ARB* angiotensin II receptor blocker, *BMI* body mass index, *CAD* coronary artery disease, *CCB* calcium channel blocker, *DOAC* direct oral anticoagulant, *ECG* electrocardiogram, *GRACE* The Global Registry of Acute Coronary Events, *HEART* history, ECG, age, risk factors, and troponin, *PAD* peripheral arterial disease, *TIA* transient ischaemic stroke, *TIMI* the thrombolysis in myocardial infarction, *VKA* vitamin K antagonist

^a^ Categorised according to the Diamond & Forrester classification

^b^ ST-segment deviation was defined as ≥ 1 mm ST-segment deviation in one or more leads

^c^ Oral anticoagulation includes DOAC and VKA

### Cardiac CT

**Fig. 2:**
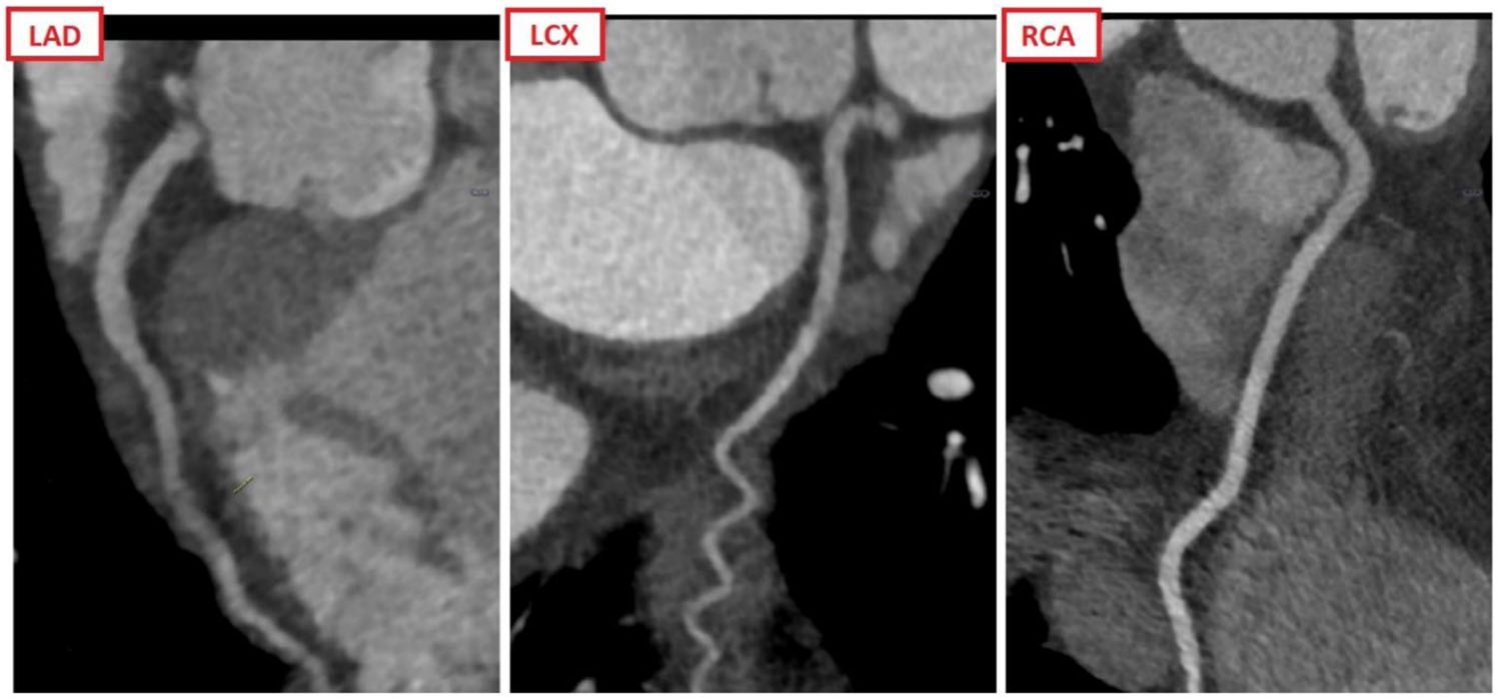
Curved multiplanar reconstructions of the three main coronary artery branches of a patient included in the COURSE trial showing no signs of coronary artery disease. Exemplary case showcasing the use of CCTA as a gatekeeper for patients with inconclusively elevated high-sensitivity troponin levels in the emergency department: an elderly patient, an active smoker, was admitted to the emergency department with typical angina. The electrocardiogram showed a previously known right bundle branch block. The first and second hs-troponin T measurements according to the European Society of Cardiology 0-h/1-h algorithm were 13 ng/L and 11 ng/L, respectively. The patient was assigned to the ‘inconclusively’ category and admitted to the Cardiology ward to undergo invasive coronary angiography. The patient was included in the COURSE trial and underwent CCTA, which showed no coronary artery disease, virtually eliminating the need for invasive coronary angiography. LAD, left anterior descending artery; LCX, left circumflex artery; RCA, right coronary artery

Cardiac CT results are presented in Table 2. All patients included in the study underwent a CAC score scan and 104 patients underwent additional CCTA. In two patients CCTA was not performed due to arrhythmia subsequent to the CAC scan. In 21 (20%) patients no CAC was present, 30 (28%) patients had a CAC score of 1–99, 17 (16%) patients had a CAC score 100–399 and finally 38 (36%) patients had a CAC score ≥ 400. Out of the 104 patients who underwent CCTA, 7 (7%) patients had a non-diagnostic scan. Of the remaining 97 patients with a diagnostic CCTA, 45 (43%) patients had a CAD-RADS score < 3 and 60 (58%) patients had a CAD-RADS score < 4A. The most common vulnerable plaque feature was positive remodelling *n* = 34 (33%), followed by evidence of low-attenuation plaque *n* = 7 (7%). The most common additional coronary finding on CCTA was myocardial bridging in 5 (5%) patients. CCTA enabled the detection of additional important non-coronary findings in 8 (8%) patients. The most common non-coronary finding was evidence of pneumonia in 4 (4%) patients. As mandated by study protocol, CCTA coronary results called for unblinding in 35 (34%) patients. Figure 2 demonstrates an exemplary case for the use of CCTA as a gatekeeper for ICA in a patient with acute chest pain and inconclusively elevated high-sensitivity troponin levels in the ED.

**Table 2:** CCTA results

	Study cohort (*n* = 104)
Heart rate	63 [56–70]
Heart-rate lowering therapy used (beta-blocker)	33 (32)
Nitroglycerine	102 (98)
Amount of iodine contrast agent in millilitre	74 [74–93]
CCTA scan mode	
Prospective	100 (96)
Flash	4 (4)
Dose length product for the entire cardiac CT procedure (in milliGray x cm)	282 [157–367]
CAD-RADS categories (degree of maximal coronary stenosis on patient-level based on CCTA)	
0 (0%)	3 (3)
1 (1–24% or plaque with no stenosis (positive remodelling))	17 (16)
2 (25–49%)	25 (24)
3 (50–69%)	15 (14)
4A (70–99%)	16 (15)
4B (Left main > 50% stenosis or 3-vessel disease)	14 (13)
5 (total occlusion/100%)	7 (7)
Non-diagnostic	7 (7)
CAD-RADS modifiers^a^	
Non-diagnostic	19 (18)
Vulnerable plaque features (one or more)	34 (33)
Positive remodelling	34 (33)
Low-attenuation plaque	7 (7)
Napkin-ring sign	0 (0)
Spotty calcification	0 (0)
Coronary findings mandating unblinding (significant left main disease, significant proximal LAD disease, significant 3-vessel disease)	35 (34)
Additional findings on CTA	
Myocardial bridging	5 (5)
Aortic dissection	1 (1)
Cardiac thrombus	1 (1)
Pneumonia	4 (4)
Pulmonary embolism	1 (1)
Malignancy	1 (1)

Values are median [interquartile ranges] or *n* (%)

*CAC* coronary artery calcium, *CCTA* coronary computed tomography angiography, *CAD-RADS* coronary artery disease reporting and data system, *cm* centimetre, *LAD* left anterior descending artery

^a^ Modifiers only contained the modifiers (1) non-diagnostic and (2) vulnerable plaque features, considering that the current study excluded patients with a history of proven coronary artery disease, defined as documented prior myocardial infarction, percutaneous coronary intervention or coronary artery bypass graft surgery

### Outcomes

**Fig. 3:**
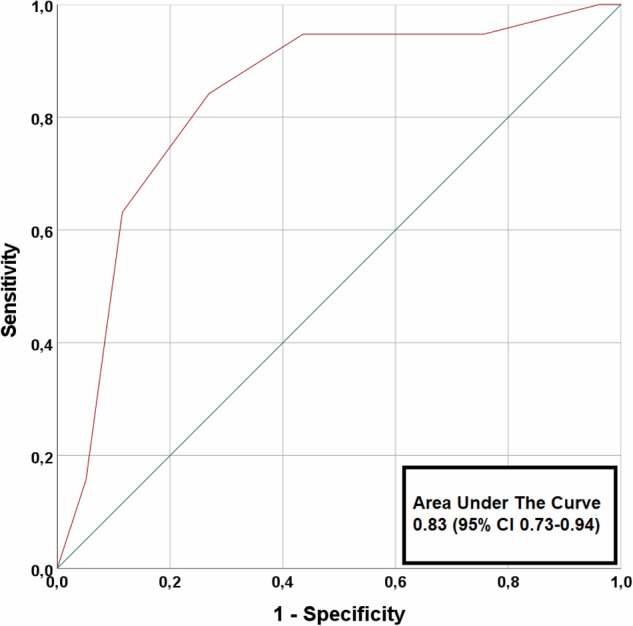
Predictive value of CCTA for type 1 NSTE-ACS. The receiver-operating-characteristic curve shows the predictive value of CCTA for type 1 non-ST-segment elevation acute coronary syndrome. CCTA, coronary computed tomography angiography

Twenty (19%) patients had an adjudicated final diagnosis of type 1 NSTE-ACS. In 75 (72%) patients CAD was observed, i.e., patients with a CAD-RADS score ≥ 1, without a clinical diagnosis of type-1-NSTE-ACS. Of these patients, only 28 (37%) patients were already on statin therapy. Table 3 shows the various diagnostic modalities used in the clinical work-up. At index visit, 43 (41%) patients were admitted to the hospital for further assessment. Within 30 days, all-cause mortality occurred in two (2%) patients and 35 (33%) patients underwent ICA, of whom 14 (13%) patients underwent coronary revascularization. In patients with non-obstructive CAD (< 50% stenosis) on CCTA, no deaths occurred, one patient had an adjudicated diagnosis of type 1 NSTE-ACS (significant stenosis in a small side branch (< 2 mm) deemed too small for intervention) and no patient underwent coronary revascularization. The AUC of CCTA for the prediction of type 1 NSTE-ACS was 0.83 (0.73–0.94) (Fig. 3). Sensitivity, specificity, NPV and PPV of ≥ 50% stenosis on CCTA to identify patients with type 1 NSTE-ACS was 95% (95% confidence interval: 74–100), 56% (45–68), 98% (87–100) and 35% (29–41), respectively. (Table 4). Sensitivity, specificity, NPV and PPV of ≥ 70% stenosis for all segments, except left main (≥ 50% stenosis) on CCTA to identify patients with type 1 NSTE-ACS was 84% (60–97), 73% (62–83), 95% (87–98) and 43% (34–54), respectively.

**Table 3:** Diagnostic testing and further management within 30 days

	Study cohort (*n* = 106)
(Non)-invasive (ischaemia) testing	
Exercise ECG	19 (18)
Echocardiography	46 (43)
SPECT	15 (14)
CMR	2 (2)
Invasive coronary angiography	35 (33)
Further management	
Admitted to hospital	43 (42)
Coronary revascularization	14 (13)
PCI	12 (11)
CABG	2 (2)

Values are *n* (%)

*CMR* cardiovascular magnetic resonance imaging, *CABG* coronary artery bypass graft surgery, *ECG* electrocardiography, *PCI* percutaneous coronary intervention, *SPECT* single-photon emission computed tomography myocardial perfusion imaging

**Table 4:** Accuracy of CCTA for the detection of type 1 NSTE-ACS

Primary outcome: ≥ 50% stenosis^a^	
Sensitivity (95% CI)	95 (74–100)
NPV (95% CI)	98 (87–100)
Specificity (95% CI)	56 (45–68)
PPV (95% CI)	35 (29–41)
≥ 50% stenosis (for the detection of type 1 NSTE-ACS, if only coronary segments with a diameter ≥ 2 mm are considered)	
Sensitivity (95% CI)	100 (81–100)
NPV (95% CI)	100 (NA)
Specificity (95% CI)	56 (45–67)
PPV (95% CI)	33 (28–38)
Secondary outcome: ≥ 70% stenosis for all coronary segments, except left main (≥ 50% stenosis)	
Sensitivity (95% CI)	84 (60–97)
NPV (95% CI)	95 (87–98)
Specificity (95% CI)	73 (62–83)
PPV (95% CI)	43 (34–54)

Values are *n* (%)

*CCTA* coronary computed tomography angiography, *CI* confidence interval, *NA* not applicable, *NSTE-ACS* non-ST-segment elevation acute coronary syndrome, *NPV* negative predictive value, *PPV* positive predictive value

^a^ Patients without CCTA scans (*n* = 2) and patients with non-diagnostic CCTA scans (*n* = 7) were excluded from the diagnostic accuracy analysis

**Fig. 4:**
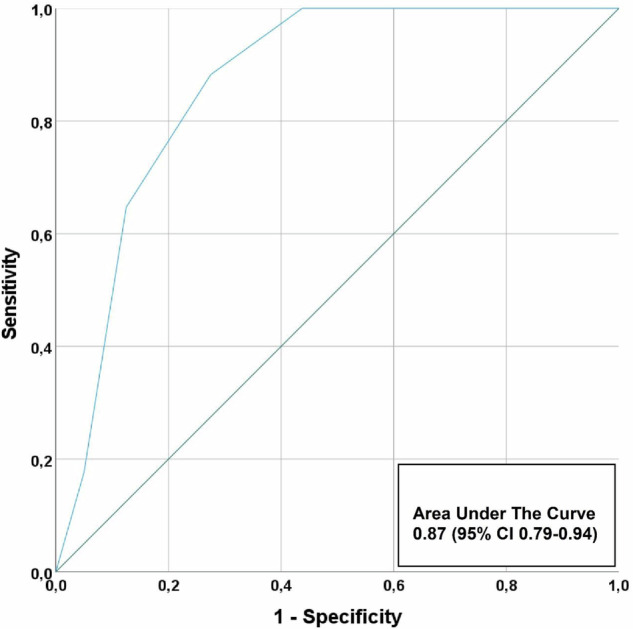
Predictive value of CCTA for type 1 NSTE-ACS, if only coronary segments with a diameter ≥ 2 mm are considered. The receiver-operating-characteristic curve shows the predictive value of CCTA for type 1 non-ST-segment elevation acute coronary syndrome if only coronary segments with a diameter ≥ 2 mm are considered. CCTA, coronary computed tomography angiography

When only coronary segments with a diameter ≥ 2 mm were considered in the adjudication process, the AUC of CCTA improved to 0.87 (0.79–0.94) (Fig. 4). Furthermore, sensitivity and NPV of ≥ 50% stenosis on CCTA to identify patients with type 1 NSTE-ACS both improved to 100% (graphical abstract).

## Discussion

In this prospective, double-blind, observational, multicentre study we examined whether CCTA can improve the diagnostic work-up of patients suspected of NSTE-ACS and inconclusively elevated hs-troponins. We report several important findings. First, ≥ 50% stenosis on CCTA is a good threshold for the rule-out of type 1 NSTE-ACS, especially when only coronary segments with a diameter ≥ 2 mm are considered. Second, CCTA enabled the detection of additional important non-coronary findings in 8% of the patients. Third, in 75 (72%) patients, CCTA enabled the detection of CAD without a diagnosis of type 1 NSTE-ACS, providing an opportunity for more strict primary prevention therapies.

The role of CCTA has previously been investigated in acute chest pain patients presenting to the ED at a time when conventional troponin assays were used [6, 10–12]. Goldstein et al compared a CCTA-based strategy to a strategy employing myocardial perfusion imaging in selected low-risk patients presenting with acute chest pain and concluded that the use of CCTA results in a more rapid and safe diagnosis than myocardial perfusion imaging [10]. Subsequently, Litt et al showed in a randomised clinical trial including low-to-intermediate risk acute chest pain patients that a CCTA-based strategy was safe and resulted in an expedited discharge as compared to standard care [11]. Based on a large clinical trial, Hoffmann et al similarly concluded that CCTA safely improved the efficiency of clinical decision-making at the ED in a low-risk acute chest pain population as compared to standard care. However, they also observed an increase in downstream testing and radiation exposure [6]. After the introduction of hs-troponins, CCTA was examined in low-risk patients with low hs-troponin values and was found not to improve clinical or logistic outcomes [13]. Over the past few years clinical evaluation of acute chest pain at the ED has markedly changed as a result of the availability of hs-troponins. Rapid rule-out and rule-in algorithms incorporating hs-troponins, e.g., The European Society of Cardiology 0/1-h algorithm, have been developed and are actively being advocated by cardiovascular societies [2, 14]. However, using these algorithms a considerable number of patients still have an inconclusive work-up after serial troponin sampling and fall into the ‘inconclusively’ category [3, 4]. These patients are known to have an unfavourable prognosis, yet the optimal diagnostic approach is unclear due to their heterogeneous nature [5]. They are burdened with prolonged observational periods at the ED or the medical ward and are exposed to potentially unnecessary invasive testing. CCTA may prove useful as a non-invasive alternative for ICA and help improve their work-up by distinguishing between a coronary and non-coronary cause for their complaints. Several studies have recently examined the value of CCTA in patients with proven NSTE-ACS and showed that CCTA can safely be used as a gatekeeper for ICA [15–17]. To the best of our knowledge, this is the first prospective study to investigate the role of CCTA specifically in patients with inconclusively elevated hs-troponins without a rise and fall pattern characteristic for myocardial infarction, i.e., patients assigned to the ‘observe group’ of the European Society of Cardiology 0/1-h algorithm.

In the current study prevalence of type 1 NSTE-ACS in the entire study population was 19%, which was comparable to the prevalence based on a previous report [5]. A stenosis of < 50% on CCTA ruled out type 1 NSTE-ACS with good sensitivity and NPV, which means that patients with non-obstructive CAD can safely forego ICA. In one patient CCTA was false negative, who was found to have a significant stenosis in a small side branch (< 2 mm) deemed too small for intervention. Therefore, missing the aforementioned patient in a ‘real-word’ setting would not directly lead to grave consequences. As opposed to ICA, CCTA also enables the detection of other cardiac and non-cardiac conditions, such as aortic dissection, pulmonary embolism and pneumonia, which may cause the presenting symptoms and thus improve the diagnostic work-up in patients with acute chest pain and inconclusively elevated hs-troponins. In our study, 8% of the patients had relevant findings, excluding myocardial bridging.

Finally, the detection of sub-clinical CAD with CCTA may open the possibility for strict primary prevention therapies in patients who fall into the ‘inconclusively’ category. Currently, European and United States guidelines already recommend the use of the CAC score in select adult patients (asymptomatic or with stable CAD) to guide preventive therapies [18, 19]. Considering that the majority of patients in this study had CAC > 100 or CAD-RADS ≥ 1, many would be a candidate for statin therapy, especially based on United States guidelines, regardless of their final diagnoses [18]. Of the 75 patients with CAD without clinical diagnosis of type 1 NSTEMI, only 28 (37%) patients were already on statin therapy due to dyslipidemia in our cohort. This means that 47 (63%) patients could still benefit from statin therapy with the aid of CCTA.

### Limitations

Due to slow inclusion, owing to restrictions on clinical research because of the global COVID-19 pandemic, and financial reasons the trial was stopped before the predefined target sample size was reached. The lower-than-expected inclusions have undoubtedly had an effect on the acquired results, e.g., the diagnostic accuracy parameters. Furthermore, this study excludes patients with known prior myocardial infarction, percutaneous coronary intervention or coronary artery bypass graft surgery, which must be taken into account when generalising the results of this study. Due to the observational nature of the study and ethical considerations, patients did not receive a standardised work-up (including ICA) and further diagnostic testing was performed at the discretion of the treating physician.

## Conclusion

In suspected NSTE-ACS patients with inconclusively elevated hs-troponins the absence of ≥ 50% coronary artery stenosis on CCTA safely and accurately rules out type 1 NSTE-ACS. Additionally, CCTA can help improve the diagnostic work-up by detecting other relevant conditions that cause acute chest pain and inconclusively elevated hs-troponins.

## Supplementary information

ELECTRONIC SUPPLEMENTARY MATERIAL

## Abbreviations

AUC: Area under the curve
CAC: Coronary artery calcium
CAD: Coronary artery disease
CAD-RADS: Coronary artery disease reporting and data system
CCTA: Coronary computed tomography angiography
ECG: Electrocardiogram
ED: Emergency department
Hs-troponin: High-sensitivity troponins
ICA: Invasive coronary angiography
NPV: Negative predictive value
NSTE-ACS: Non-ST-segment elevation acute coronary syndrome
PPV: Positive predictive value
