# Supplementary material for: Coronary computed tomography angiography improves assessment of patients with acute chest pain and inconclusively elevated high-sensitivity troponins
Source: Eur Radiol. 2024 Aug 16;35(2):789–97. doi: 10.1007/s00330-024-10930-1 (PMC11782329; doi:10.1007/s00330-024-10930-1)
Supplement: Supplementary file 1 — ELECTRONIC SUPPLEMENTARY MATERIAL [file 330_2024_10930_MOESM1_ESM.pdf]

Coronary Computed Tomography Angiography Improves Assessment of Patients with Acute Chest Pain and Inconclusively Elevated High-Sensitivity Troponins

ELECTRONIC SUPPLEMENTARY MATERIAL

Supplementary table 1. Additional information on CT scanners used and CCTA protocols in the Course trial

Vendor/scanner type	**Canon Acquilon One Prism 0.5 mm x 320 rows**	**Siemens Somatom Force Single source**	**Siemens Somatom definition FLASH dual source**
Acquired slice collimation	0.5 mm	0.6 mm	0.6 mm
Tube voltage (kV)	Auto kV (Reference kV 80/100/120)	Auto kV(care kV on) Reference kV 120	Auto kV(care kV on) Reference kV 120
Tube current (mAs)	SD 40 Minimum 40 mA	CareDose 4D on Reference mAs 115	CareDose 4D on Reference mAs 320
Image reconstruction	Volume 1: CTA: AiCE Cardiac 0.5/0.25. Volume 2: PhaseXact Best Phase: AiCE Cardiac 0.5/0.25	ADMIRE	ADMIRE
Slice thickness	0.5 mm	0.6 mm	0.6 mm
Slice increment	0.5/0.25 mm	0.4 mm	0.4 mm
Kernel	AiCE Cardiac	Bv40	Bv40
Usage of post processing software	AiCE Body Sharp op 1/1. TeraRecon iNtuition 4.6.1.48	Siemens Healthineers, syngo.via	Siemens Healthineers, syngo.via
ECG timing	- Heart frequency <65; RR – interval 70% - 80%<br>- Heart frequency 65 – 100; RR – interval 30% - 80%<br>- Heart frequency >100; RR – entire RR interval	- Heart frequency <65; RR – interval 62% - 77%<br>- Heart frequency 70 – 95; RR – interval 33% - 77%	- Heart frequency <65; RR – interval 65% - 75%<br>- Heart frequency 65 – 80; RR – interval 35% - 75%<br>- Heart frequency >80; RR – interval 35% - 55%

ECG= electrocardiogram; kV=kilovoltage; mAs=Milliampere-seconds; mm=millimeter.
